# Enhancing the high-voltage electrochemical performance of LiNi_0.8_Co_0.1_Mn_0.1_O_2_ cathodes through La_0.2_Sr_0.8_TiO_3_ surface coating

**DOI:** 10.1039/d6ra03778j

**Published:** 2026-07-28

**Authors:** Song Sun, Shengjie Zhou, Jiawei Wu, Meixin Huang, Yu Li, Sujiao Lan, Guanhua Yang

**Affiliations:** a Department of Food and Chemical Engineering, Liuzhou Institute of Technology Liuzhou 545616 P.R. China liyu-306@163.com; b Guangxi Key Laboratory of Automobile Components and Vehicle Technology, School of Mechanical and Automotive Engineering, Guangxi University of Science and Technology Liuzhou 545006 P.R. China yghchem@163.com

## Abstract

The highly active surface of LiNi_0.8_Co_0.1_Mn_0.1_O_2_(NCM811) cathodes frequently causes rapid surface degradation, leading to diminished cycling and rate capabilities, particularly under high voltage conditions exceeding 4.5 V. Herein, we report an approach to boost the electrochemical performance of NCM811 through integrated surface engineering. The highly conductive La_0.2_Sr_0.8_TiO_3_(LSTO) acts as an effective protective layer to mitigate interfacial side reactions. Moreover, this coating strategy prevents the additional impedance commonly induced by standard surface treatments. After 300 cycles at 1C, the optimized N-LSTO2 cathode material exhibits a remarkably enhanced capacity retention of 88.19%, whereas the pristine NCM811 only maintains 38.73%. Additionally, the N-LSTO2 cathode exhibits a discharge capacity of 120.3 mAh g^−1^ at 10C rate, which significantly outperforms the 89.6 mAh g^−1^ observed for the pristine cathode. The enhanced electrochemical properties originate from the highly conductive LSTO protective barrier, which creates an amorphous layer of approximately 4 nm in thickness on the NCM811 surface. The effective separation between NCM811 and the electrolyte provided by this coating leads to the inhibition of severe interfacial side reactions, coupled with enhanced electron transfer kinetics at the electrode/electrolyte interface.

## Abstract 2

A multifunctional La_0.2_Sr_0.8_TiO_3_ coating effectively shields NCM811 from HF attack and facilitates electron transport, unlocking exceptional long-term cycling stability.

## Graphical abstract

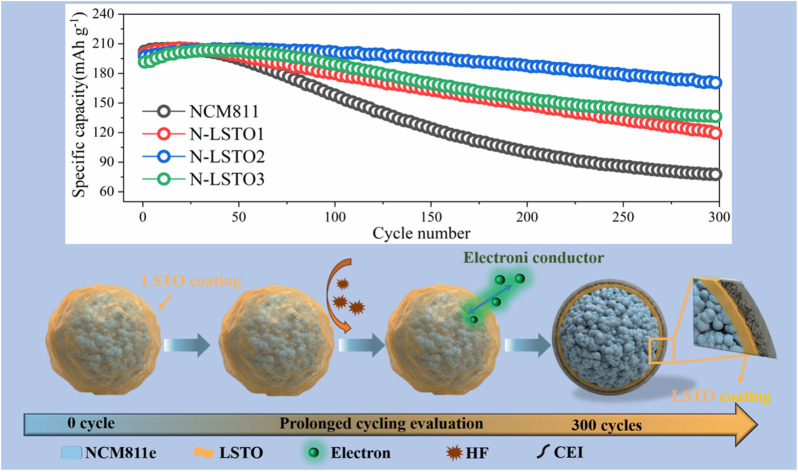

## 1. Introduction

Lithium-ion batteries (LIBs) stand out among various energy storage devices as the optimal power solution for electric and hybrid electric vehicles (EVs/HEVs), primarily driven by their high energy density and low weight.^1,2^ However, the development of long-range EVs demands LIBs with superior energy and power densities. Driven by their high capacity, elevated operating voltage, and cost-effectiveness, Ni-rich layered structure materials have emerged as the most viable cathode candidates for high-energy-density LIBs.^3–5^ Especially, LiNi_0.8_Co_0.1_Mn_0.1_O_2_ (NCM811), which possesses a hexagonal lattice with an *R*3̄*m* space group analogous to the α-NaFeO_2_ layered structure, and has drawn extensive attention recently due to its impressive reversible capacity of ∼200 mAh g^−1^.^6^ However, the structural integrity of NCM811 is severely compromised by the irreversible migration of Ni into the Li layer upon deintercalation. This phenomenon, prevalent under high-voltage operation, results in deleterious phase transitions from a layered (*R*3̄*m*) to spinel-like (*Fd*3̄*m*) and rock-salt (*Fm*3̄*m*) configurations.^7,8^ Additionally, the residual lithium species on the NCM811 surface readily react with ambient CO_2_ and H_2_O. Accompanied by surface deoxygenation, these reactions generate a defective resistive overlayer, which can be elucidated by the following equations: Ni^3+^ + O^2−^ → Ni^2+^ + O^−^, O^2−^ + CO_2_/H_2_O → CO2_2–3_/2OH^−^, 2Li^+^ + CO2_2–3_/2OH^−^ → Li_2_CO_3_/2LiOH.^9,10^ These phenomena on the material surface accelerate negative side effects upon direct contact with the electrolyte, leading to deterioration of electrochemical performance during electrode processes. Furthermore, due to the attack by hydrofluoric acid, the dissolution of transition metals in the liquid electrolyte is another detrimental byproduct that results in poor cycling and rate capabilities.^11,12^ Multiple approaches have been taken to address these issues, such as ion doping, surface modification, and adding functional electrolyte additives.^13–15^

Surface engineering with a thin coating layer provides a workable solution to the above challenges: acting as a physical shield, this layer keeps Ni-rich material from directly touching the electrolyte, thus stopping unwanted interfacial reactions. Up to now, a variety of materials, including conductive oxides, reduced graphene oxide, carbon, fluorides, and phosphates, have been utilized as surface coatings and interfacial stabilization layers, which have demonstrated effectiveness in inhibiting surface side reactions, thereby enhancing the overall electrochemical performance of Ni-rich layered oxides.^16,17^ However, conventional inert oxides, fluorides, and phosphates typically lack electronic conductivity, which exacerbates electrode polarization. Meanwhile, carbon-based coatings often hinder lithium-ion diffusion and risk surface reduction of the Ni-rich cathode during preparation. For this reason, using high ion conductivity coatings is seen as an important way to get the desired cycling stability and rate capability. The inorganic Li-based oxides such as Li_3_PO_4_, Li_2_ZrO_3_, LiAlO_2_, Li_4_SiO_4_, and Li_1.3_Al_0.3_Ti_1.7_(PO_4_)_3_ are extensively being explored as coating layers to facilitate fast Li^+^ diffusion on the cathode surface.^18–20^ Nevertheless, reducing process complexity and obtaining coating materials with optimal structural compatibility with the cathodes remain key challenges that need to be addressed. Furthermore, while traditional inorganic coatings primarily address ionic transport, mitigating the severe electrode polarization of NCM811 at high voltages additionally necessitates a robust electronic conductive network.^21,22^ As an exceptional electronic conductor, La_0.2_Sr_0.8_TiO_3_(LSTO) is renowned for its superior electronic conductivity, wide electrochemical window, and excellent catalytic properties.^23,24^ Compared to traditional coatings, LSTO provides a synergistic effect by combining robust physical protection against interfacial reactions with an efficient electronic network to accelerate charge transfer. Owing to these exceptional properties, it has emerged as a highly attractive electrode candidate for solid oxide fuel cells.^25^ Building upon the aforementioned research, LSTO is selected to construct a highly conductive layer to enhance the high-voltage performance of the NCM811 cathode. Specifically, the material's exceptional electrical conductivity is expected to enhance the kinetics of lithium-ion surface intercalation, reduce electrode polarization, and decrease interfacial resistance between the active bulk and the liquid electrolyte.

This study successfully prepared an LSTO-coated NCM811 cathode using a sol–gel technique. The fundamental novelty of our approach lies in constructing a unique nanocrystalline-in-amorphous hybrid LSTO layer on the cathode surface. Unlike highly crystalline conductive oxides, this specific structural state transforms the conformal LSTO coating into a highly efficient hybrid ion-electron conductor. The LSTO layer provides a comprehensive synergy effect: on the one hand, it acts as a strong physical barrier, reducing electrode–electrolyte contact and inhibiting adverse reactions; on the other hand, it can improve the transmission rate of Li^+^ and electrons on materials. As a result, the 2% LSTO-coated NCM811 cathode achieves a capacity retention rate of 88.19% after 300 cycles between 2.75 and 4.5 V at 1C, and delivers 120.3 mAh g^−1^ at 10C.

## 2. Experimental

### 2.1 Preparation of LSTO-modified NCM811 materials

The pristine NCM811 cathode was prepared *via* a standard co-precipitation technique coupled with subsequent thermal annealing. Initially, the Ni_0.8_Co_0.1_Mn_0.1_(OH)_2_ precursors was precipitated from a stoichiometric mixture of nickel, cobalt, and manganese sulfates. After thoroughly homogenizing this precursor with LiOH·H_2_O at a molar ratio of 1 : 1.05, the mixture was calcined in an oxygen atmosphere. Subsequently, it underwent sintering at 480 °C and 850 °C under oxygen flow for 6 and 12 h, respectively. Finally, the final pristine NCM811 powder was collected upon natural cooling to ambient temperature.

The NCM811@LSTO cathode was fabricated using a sol–gel approach coupled with a high-temperature solid-state reaction. Specifically, the LSTO coating was prepared using analytical-grade La(NO_3_)_3_·6H_2_O, Sr(NO_3_)_2_, and titanium isopropoxide as precursors, dissolved in an ethanol solvent with citric acid acting as the chelating agent. Firstly, La(NO_3_)_3_·6H_2_O, Sr(NO_3_)_2_, and citric acid were dissolved in 30 ml of ethanol according to the stoichiometric ratio of the coating layer LSTO, and then 2 g of NCM811 cathode material was added and stirred for 30 min. Secondly, under stirring, titanium isopropoxide was slowly added dropwise into the above beaker, and after the dropwise addition was completed, stirring was continued for 2 h, followed by drying at 80 °C. Finally, the as prepared sample was placed in a tubular furnace, heated in air at a rate of 3 °C min^−1^ to 600 °C for 4 h, then naturally cooled to room temperature to obtain the NCM811@LSTO composite electrode material. Samples with different coating amounts were prepared according to the mass ratio of the coating layer LSTO to NCM811 at 1 wt%, 2 wt%, and 5 wt%, which are denoted as N-LSTO1, N-LSTO2, and N-LSTO3, respectively.

### 2.2 Material characterizations

To elucidate the crystal structures of the pristine NCM811 and LSTO-coated cathodes, X-ray diffraction (XRD) measurements were performed using a Rigaku D/Max-2500 V/PC powder diffractometer equipped with a Cu Kα radiation source (*λ* = 0.15406 nm, Rigaku, Japan). The surface morphological examinations and elemental analyses were conducted using a Philips FEI Quanta 200 FEG scanning electron microscope (SEM) equipped with an energy-dispersive X-ray spectrometer (EDS). Meanwhile, precise lattice spacing measurements were achieved *via* high-resolution transmission electron microscopy using a Philips FEI Tecnai G2F30 instrument. Surface composition and valence state analyses relied on X-ray photoelectron spectroscopy (XPS) performed with a Thermo Scientific Escalab 250 XI instrument equipped with a monochromatized Al Kα X-ray source (1486.6 eV). The survey spectrum was acquired with a pass energy of 150 eV and a step size of 1.0 eV, while the high-resolution spectra were collected with a pass energy of 50 eV and a step size of 0.1 eV. During the measurements, a dual-beam charge neutralisation system (using low-energy electrons and Ar^+^ ions) was continuously used to compensate for the surface charge effect, and all measurements were carried out under ultra-high vacuum conditions.

### 2.3 Electrochemical testing

The electrochemical behavior of the as-prepared samples was evaluated in CR2032 coin-type half-cells. For the electrode preparation, a mixture of 85 wt% active material, 5 wt% super-P carbon black, 5 wt% KS-6, and 5 wt% polyvinylidene fluoride binder was homogeneously dispersed in *N*-methyl-2-pyrrolidinone. The slurry was cast onto aluminum foil current collectors and initially dried. Then, the coated foil was punched into 12 mm diameter disks and dried under vacuum at 120 °C for 12 h. The active material mass loading of each electrode was controlled to be approximately 2.5–3 mg cm^−2^, and the total electrode areal density (including active material, conductive carbon, and PVDF binder) was approximately 2.94–3.53 mg cm^−2^. The CR2032 coin cells was carried out inside an argon-filled glovebox, utilizing lithium metal anodes, polypropylene separators, and a commercial electrolyte comprising 1 M LiPF_6_ dissolved in a 1 : 1 : 1 (v/v/v) solvent blend of ethylene carbonate, dimethyl carbonate, and ethyl methyl carbonate (supplied by Shanghai JingShong Material Co., Ltd). Galvanostatic charge–discharge tests were performed on a LAND battery testing system operating between 2.75 and 4.5 V at various C-rates (1C = 200 mA g^−1^). Furthermore, electrochemical impedance spectroscopy (EIS) measurements were conducted using a Zahner IM6 workstation (Zahner-Elektrik GmbH & Co. KG, Germany) by applying a 5 mV AC perturbation across the frequency range of 0.01 Hz to 100 kHz.

## 3. Results and discussion

**Fig. 1:**
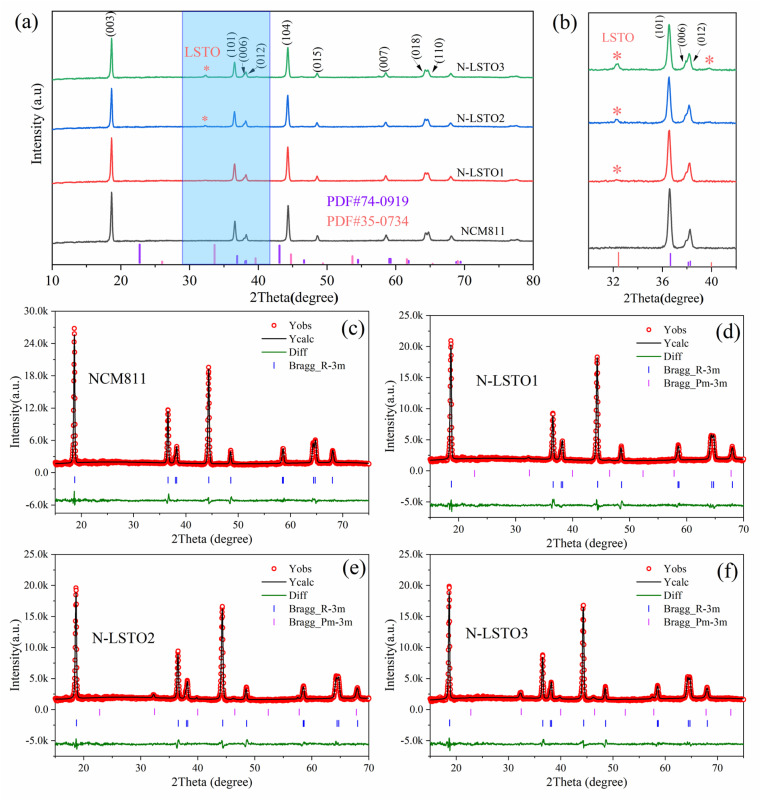
(a) XRD patterns of NCM811 and LSTO-coated with different coating amounts of LSTO; (b) a magnified view of (a). (c–f) The Rietveld refinement patterns of NCM811 and LSTO-coated with different coating amounts of LSTO, respectively.

Fig. 1a displays the XRD patterns of the pristine and LSTO-coated NCM811 samples. All diffraction peaks of these samples can be well indexed to the layered hexagonal α-NaFeO_2_ structure of the *R*3̄*m* space group.^26^ The evident peak splitting of the (006)/(102) and (108)/(110) pairs in the patterns suggests a well-ordered layered crystal structure. Furthermore, as the coating content increases to 2 wt%, a small peak at around 32.5° can be distinctly observed, which corresponds to LSTO (PDF # 35-0734). With further increases in the coating content, the diffraction peaks of LSTO become more pronounced, particularly in the N-LSTO3 sample as depicted in the enlarged view of Fig. 1b, while the diffraction peaks of LSTO for the N-LSTO1 sample are not prominent, likely due to its minimal coating concentration. The detection of these peaks by XRD, a bulk characterization technique, indicates the formation of a minor crystalline LSTO phase at 600 °C when the coating content is sufficient. To acquire detailed structural parameters, Rietveld refinement was conducted, as presented in Fig. 1c–f. The calculated profiles match well with the observed data, and the Bragg tick marks conclusively establish the coexistence of the predominant layered NCM811 phase and the secondary cubic LSTO phase in the engineered samples. To quantitatively evaluate the structural changes, the detailed Rietveld refinement parameters are summarized in Table S1. As shown in Table S1, the *c*/*a* ratios for all samples are greater than 4.9, indicating a well-ordered layered structure. Furthermore, the *I*_(003)_/*I*_(104)_ ratios are all above 1.2, which signifies a well-ordered layered structure is maintained with a low degree of cation mixing of both samples. These findings further demonstrate that the elements from the LSTO coating do not penetrate the host lattice structure, thereby preserving the intrinsic integrity of the well-crystallized NCM811 grains.

**Fig. 2:**
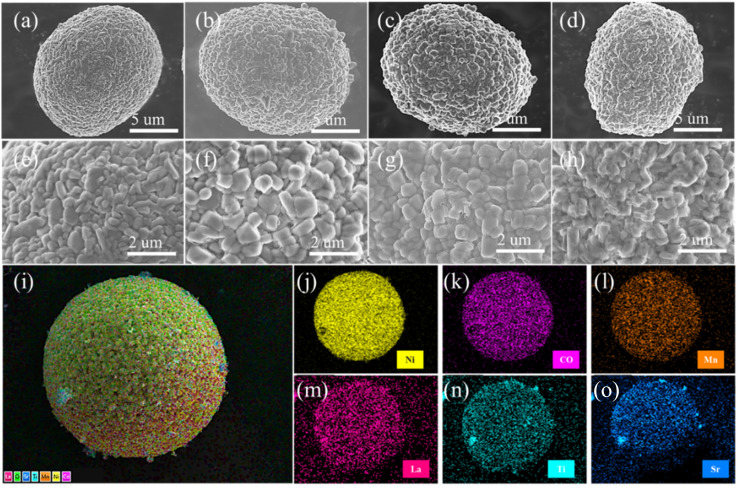
SEM images of (a and e) pristine NCM811, (b and f) N-LSTO1, (c and g) N-LSTO2, and (d and h) N-LSTO3. (i) Overlay EDS mapping image of the N-LSTO2 powder, and corresponding elemental mappings for (j) Ni, (k) Co, (l) Mn, (m) La, (n) Ti, and (o) Sr.

The surface morphologies of the pristine NCM811, N-LSTO1, N-LSTO2, and N-LSTO3 samples were initially investigated *via* SEM, as presented in Fig. 2. Both the pristine NCM811 and the LSTO-coated NCM811 materials exhibited spherical secondary particles approximately 10 µm in diameter, composed of agglomerated nanoparticles ranging from 50 to 80 nm. While the overall macro-morphology of the secondary particles shows negligible differences with the introduction of LSTO, noticeable changes occur at the microscopic surface level. The pristine NCM811 exhibits a smooth surface. In contrast, the surfaces of all LSTO-modified samples become noticeably rougher, which can be attributed to the successful deposition of LSTO nanoparticles. Additionally, the surface roughness becomes more pronounced with increasing LSTO content. The overlay EDS mapping images (Fig. 2i) and the individual elemental maps (Fig. 2j–o) reveal that Ni, Co, Mn, La, Sr, and Ti elements are distributed homogeneously across the N-LSTO2 sample, confirming the uniform coating of LSTO onto the NCM811 particles.

**Fig. 3:**
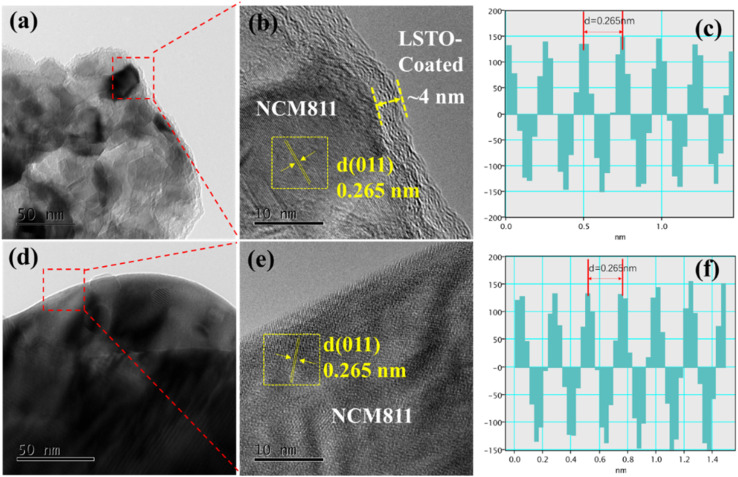
(a) The TEM image of N-LSTO2, (b) the HR-TEM image of N-LSTO2 magnified view of the red region in (a); (c) the corresponding line-scan intensity profile of the lattice fringes in (b); (d) the TEM image of NCM811; (e) the HR-TEM image of NCM811 magnified view of the red region in (d); (f) the corresponding line-scan intensity profile of the lattice fringes in (e).

To further investigate the microstructures of the pristine NCM811 and N-LSTO2 samples, TEM and HRTEM analyses were performed and the detail were presented in Fig. 3. The Fig. 3b shows a clear amorphous layer on the NCM811 matrix surface, which matches the LSTO coating layer first shown in Fig. 3a. It should be emphasized that the LSTO coating is predominantly an amorphous layer with a thickness of approximately 4 nm (Fig. 3b), although a small amount of crystalline LSTO phase coexists in the bulk sample as detected by XRD. The predominantly amorphous nature observed in HRTEM is due to the relatively low calcination temperature (600 °C) and the localized nanoscale observation area. Furthermore, the internal region exhibits clear lattice fringes with a *d*-spacing of 0.265 nm corresponding to the (011) plane, as validated by the line-scan intensity profile in Fig. 3c. In contrast, the surface of the pristine sample is smooth and clean, markedly distinct from that of the modified sample, as illustrated in Fig. 3d and the corresponding HRTEM image in Fig. 3e. The lattice fringes of the pristine sample also display an identical *d*-spacing of 0.265 nm (Fig. 3f). This demonstrates that LSTO has effectively coated the surface of the NCM811 material without altering the intrinsic crystal structure of the bulk material.

**Fig. 4:**
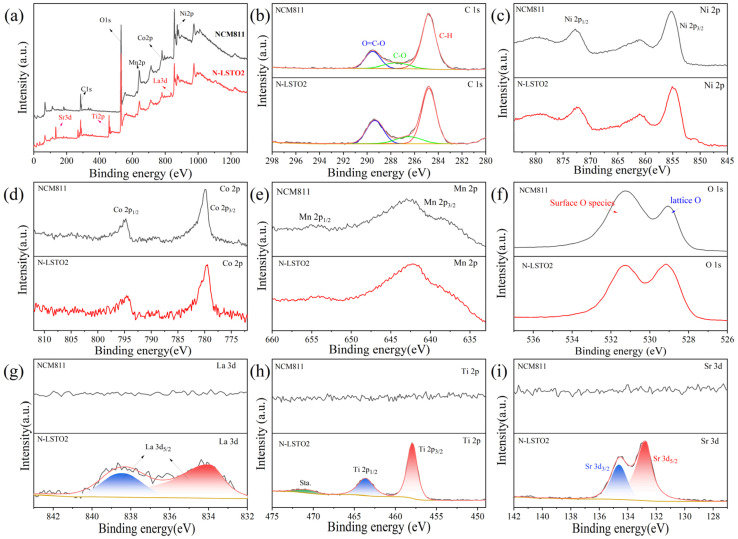
XPS survey spectra (a), and high-resolution XPS spectra of C 1s (b), Ni 2p (c), Co 2p (d), Mn 2p (e), O 1s (f), La 3d (g), Ti 2p (h), and Sr 3d (i) elements of the pristine NCM811 and N-LSTO2 samples.

The chemical composition and oxidation states of the surface elements for both the pristine NCM811 and N-LSTO2 samples were analyzed using X-ray photoelectron spectroscopy (XPS), with peak deconvolution performed by the XPSPEAK software. As shown in the full survey spectra (Fig. 4a), the N-LSTO2 sample presents distinct La 3d, Ti 2p, and Sr 3d peaks compared to the pristine NCM811, which provides direct evidence for the successful introduction of the LSTO coating layer.^27^ As shown in Fig. 4c–e, the high-resolution XPS spectra of Ni 2p, Co 2p, and Mn 2p are presented to evaluate the local chemical environment of the transition metals. By comparing the overall binding energy shifts, it can be observed that the chemical environment of the transition metals in the NCM lattice remains essentially unchanged after LSTO modification, as evidenced by the negligible binding energy shifts observed in the Ni 2p, Co 2p, and Mn 2p spectra of the N-LSTO2 sample relative to the pristine sample. Furthermore, the high-resolution spectra of the specific coating elements are depicted in Fig. 4g–i. The La 3d spectrum (Fig. 4g) exhibits the characteristic La 3d_5/2_ and La 3d_3/2_ peaks. The Ti 2p spectrum (Fig. 4h) and Sr 3d spectrum (Fig. 4i) confirm the typical Ti^4+^ and Sr^2+^ states, respectively. Additionally, the C 1s (Fig. 4b) was used to identify the carbon-containing species on the surface. For the high-resolution O 1s spectrum (Fig. 4f), the peak at about 529.5 eV corresponds to lattice oxygen, while the 531.0–532.0 eV region corresponds to surface oxygen species, specifically including chemisorbed oxygen, carbonates, and hydroxyl groups. Through the qualitative comparison of the binding energy positions and the overall spectral features, it can be clearly seen that the lattice oxygen framework of the NCM811 remains essentially unchanged after LSTO coating.^28–30^

**Fig. 5:**
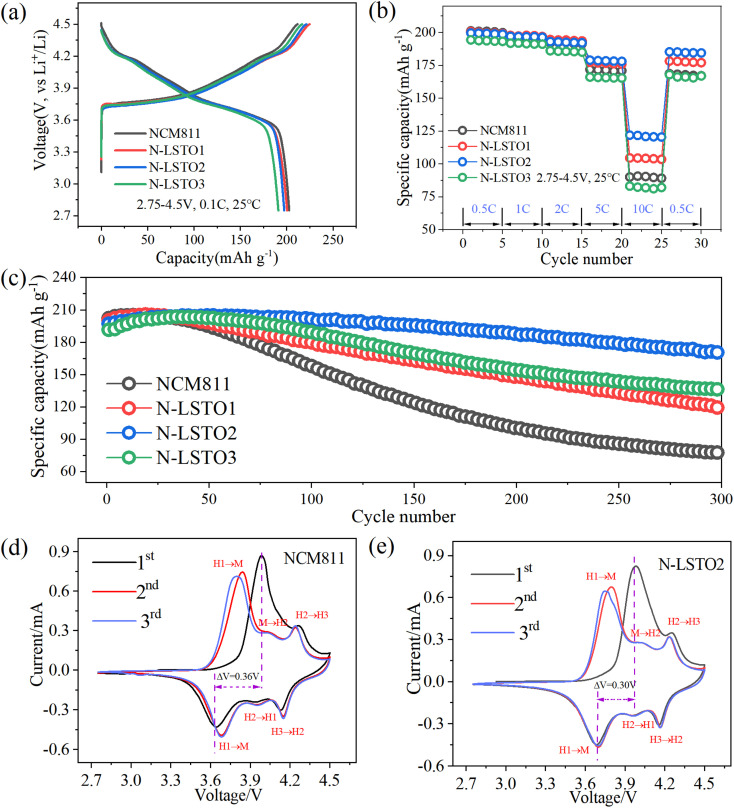
(a) Galvanostatic charge–discharge profiles, (b) rate metrics, and (c) room-temperature cycling stability at room temperature of the pristine NCM811 and N-LSTO-coated samples; CV profiles of (d) the pristine NCM811 and (e) N-LSTO2 samples for the first three cycles.

The electrochemical performances of the pristine NCM811 and LSTO-coated samples were evaluated to demonstrate the advantages of the surface modification. Galvanostatic charge and discharge test was conducted under a constant current of 0.1C (1C = 200 mA g^−1^) between 2.75 and 4.5 V at room temperature and the profiles are shown in Fig. 5a. Though initial curves have similar sloping voltage features, there are clear differences in specific capacities and coulombic efficiencies for both samples. The pristine NCM delivers a superior initial discharge capacity of 204.7 mAh g^−1^ and a coulombic efficiency of 91.10%, whereas these metrics progressively decline in the coated samples as the LSTO content increases. For the N-LSTO samples with varying coating amounts, the discharge capacities are 201.5, 197.3, and 191.1 mAh g^−1^, and the corresponding efficiencies of 89.64%, 89.11%, and 88.15%, respectively. Initial irreversible capacity loss mainly comes from solid electrolyte interphase (SEI) film formation; slightly lower specific capacities of coated electrodes are because electrochemically inactive LSTO mass is included in total weight calculations. It should be noted that the introduction of LSTO coating inevitably involves a trade-off between the initial electrochemical properties and long-term cycle stability. Although the electrochemically inert coating will lead to a slight decrease in the first discharge ratio capacity and the first Coulomb efficiency, it can also effectively improve the structural stability and interface stability of the positive material in the long-term cycle. Therefore, for the evaluation of the effectiveness of the coating strategy, we should not only focus on the initial energy density, but also consider its long-term cycle life and capacity retention ability. The electrochemical performance of the LSTO-coated NCM811 is compared with representative coated NCM811 systems reported in recent years, as summarized in Table S2. After activation at 0.1C for 2 cycles, room-temperature cycling performances at 1C were evaluated, as shown in Fig. 5c. After 300 cycles, N-LSTO1, N-LSTO2, and N-LSTO3 cathodes have discharge capacities of 119.7, 170.3, and 136.4 mAh g^−1^, with corresponding capacity retentions of 60.98%, 88.19%, and 72.75% respectively. The better capacity retention of N-LSTO2 demonstrates that an optimal LSTO coating amount works as a strong interfacial shield against electrolyte effects. Furthermore, to evaluate the practical applicability of the proposed surface modification strategy, additional electrochemical tests were performed under a higher areal mass loading of approximately 6.0–6.5 mg cm^−2^, which is more than twice that used in the main manuscript (2.5–3.0 mg cm^−2^). As shown in Fig. S1, although the overall specific capacity is reduced due to the increase in polarization and the slowdown of Li^+^ transport associated with thick electrodes, the N-LSTO2 electrode still shows significantly better cycling stability than the pristine sample after 150 cycles. These results demonstrate that the beneficial effect of the LSTO coating is maintained under conditions closer to practical electrode configurations, further highlighting the practical application potential of this surface modification strategy for lithium-ion batteries.

Cyclic voltammetry (CV) measurements were performed to further study the electrochemical kinetics. Fig. 5d and e show that N-LSTO2 has a potential difference (Δ*V*) of 0.30 V compared to 0.36 V for the pristine NCM811. This means pristine NCM811 has larger polarization and weaker electrochemical reversibility than N-LSTO2. To further understand the reaction kinetics and charge storage behavior, we conducted CV measurements at scan rates ranging from 0.1 to 0.8 mV s^−1^ (Fig. S2). As the scan rate increases, the redox peak currents of both electrodes gradually increase. In addition, the peak current (*I*_p_) exhibits an approximately linear relationship with the square root of the scan rate (*v*^1/2^), indicating that the electrochemical reactions of both electrodes are mainly governed by a diffusion-controlled Li^+^ intercalation/deintercalation process. It is worth noting that, compared with pristine NCM811 (*k* ≈ 3.175), the N-LSTO2 electrode exhibits a significantly higher fitted slope (*k* ≈ 4.211). The larger slope suggests enhanced Li^+^ diffusion kinetics and improved charge-transfer behavior in the N-LSTO2 electrode. This result further confirms that the LSTO coating layer not only effectively stabilizes the electrode/electrolyte interface but also promotes electrochemical reaction kinetics, thereby facilitating Li^+^ transport during cycling.

**Fig. 6:**
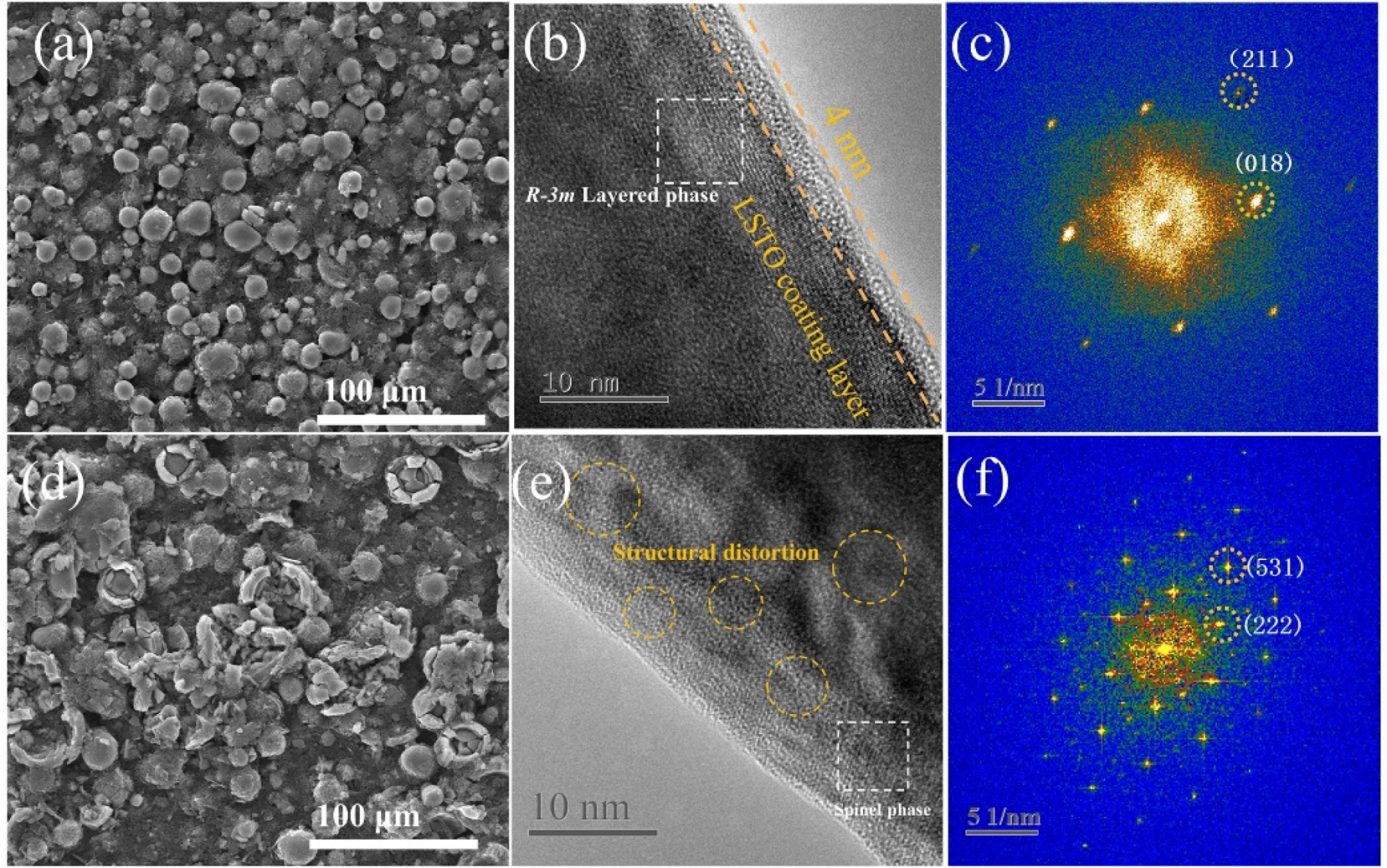
The morphological and structural characterizations of the electrodes after 300 cycles at 25 °C. (a) SEM, (b) HRTEM, and (c) the corresponding FFT pattern of the N-LSTO2 sample. (d) SEM, (e) HRTEM, and (f) the corresponding FFT pattern of the pristine NCM811 sample.

Fig. 5b shows rate capabilities tested to assess electrochemical kinetics of both samples. The pristine NCM811 has the highest initial capacity at low current densities among the four samples, it has severe capacity decay at higher rates, which only retaining 89.6 mAh g^−1^ at 10 C. In contrast, the N-LSTO2 shows excellent high-rate tolerance, keeping an average discharge capacity of about 120.3 mAh g^−1^ even at 10 C. Fig. 5b illustrates the rate capabilities of the investigated electrodes to assess their electrochemical kinetics. Although the pristine NCM811 delivering the highest initial capacity at low current density in the four samples, it experienced severe capacity decay at high magnification, retaining only 89.6 mAh g^−1^ at 10 C. On the contrary, the N-LSTO2 sample showed excellent high rate capability and could maintain an excellent average discharge ratio capacity of about 120.3 mAh g^−1^ at 10 C. These results show that the an optimal LSTO coating amount is crucial to significantly improve the rate capability, as evidenced by the performance of N-LSTO1 and N-LSTO2 samples. In addition, the specific capacity of the N-LSTO3 electrode has been significantly inhibited due to the excessive coating layer. This degradation of electrochemical kinetics can be attributed to the increased interface impedance of the too thick LSTO shell, thus greatly prolonging the diffusion path of Li^+^ from the electrolyte into the active body. NCM811 cathodes usually undergo severe structural degradation throughout extended cycling protocols. To further investigate the structural stability, SEM and TEM were performed on electrodes cycled for 300 cycles at 25 °C. SEM image of the pristine NCM811 shows evident cracks and severe pulverization in secondary particles (Fig. 6d). Dramatically, the cracks can be found elsewhere in the SEM. This feature suggests that the pristine sample experienced serious nanostrain during battery operation. When evaluating the TEM images and corresponding FFT patterns, severe structural distortion and a spinel phase with (531) and (222) planes are observed (Fig. 6e and f). By contrast, the N-LSTO2 displays negligible structural degradation during the 300 cycles. After cycling, the N-LSTO2 sample maintained a lower interface impedance and a more complete structure, indicating that the LSTO coating layer contributed to improved structural stability and interfacial integrity during prolonged cycling. Fig. 6a shows a highly intact spherical morphology without obvious cracks on the N-LSTO2 secondary particle. TEM images and corresponding FFT patterns also show an ordered *R*3̄*m* layered structure with distinct (211) and (018) planes, and amorphous LSTO (Fig. 6b). The introduction of LSTO reduces the surface energy and interfacial side reactions, thereby promoting the structural integrity of secondary particles. This process, in turn, mitigates stress concentration and suppresses crack propagation during cycling.

To further corroborate the suppression of interfacial degradation, post-cycling XPS analysis was conducted after 150 cycles (Fig. S3). The quantitative results strongly support the protective effect of LSTO coating. In the C 1s spectrum, the proportion of O–C=O peak (289.5 eV, representing organic carbonate by-products) decreased significantly from 15.3% of the original sample to 6.8% of the N-LSTO2 sample, indicating that solvent oxidation is effectively inhibited. Similarly, the P 2p spectrum shows a significant decrease in the proportion of P–O peaks (from 58.9% to 29.1%), confirming that serious LiPF_6_ salt decomposition is inhibited. More importantly, in the O 1s spectrum, compared with the original positive pole (26.3%), the positive pole covered by LSTO shows a relatively higher proportion of the lattice oxygen signal (30.2%). Considering the limited detection depth of XPS, this strong lattice oxygen signal directly proves that the mixed LSTO coating limits the continuous interface parasitic reaction, thus promoting the formation of an exceptionally thin, dense, and stabilized CEI layer over prolonged cycling. Furthermore, to further investigate the crack evolution behavior during cycling, SEM observations were performed after 0, 50, 100, and 150 cycles (Fig. S4). As the number of cycles increased, the crack density and degree of particle fragmentation in the pristine NCM811 electrode gradually increased, indicating that structural degradation progressively accumulated during cycling rather than occurring during the initial cycles. In contrast, the N-LSTO2 electrode maintained a relatively intact particle morphology after prolonged cycling, indicating that the LSTO coating layer can effectively suppress crack propagation and improve structural stability (Fig. 6). These observations are consistent with the improved interfacial stability revealed by the post-cycling XPS analysis.

**Fig. 7:**
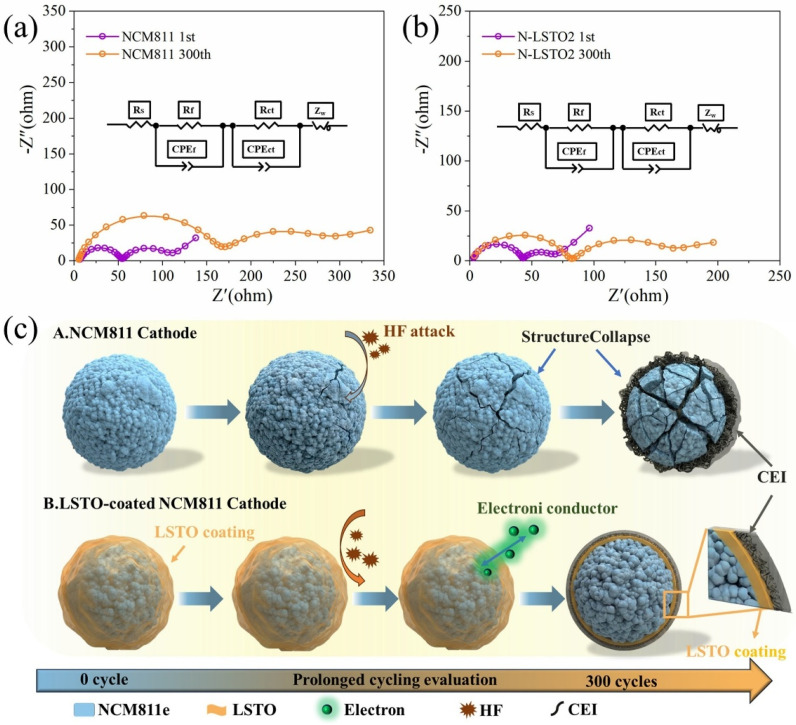
EIS profiles and the corresponding equivalent circuit models of (a) the pristine NCM811 and (b) N-LSTO2 electrodes after the 1st and 300th cycles. (c) The Schematic illustration of the structural evolution and interfacial stabilization mechanism of the pristine and LSTO-coated NCM811 cathodes during prolonged cycling.

To elucidate the impact of the LSTO modification on Li^+^ diffusion kinetics and interfacial properties, EIS measurements were conducted. The impedance spectra of the cells containing pristine NCM811 and N-LSTO2 cathodes were recorded after the initial activation cycle and following 300 cycles, as displayed in Fig. 7a and b, respectively. Fig. 7 presents the Nyquist plots along with the corresponding equivalent circuit model, which was refined *via* ZSimpWin software. The plots comprise two consecutive depressed semicircles spanning the high-to-medium frequency domains, culminating in a sloping Warburg impedance (*Z*_W_) line at low frequencies. Although there are obvious cracks in the pristine NCM811 electrode after cycling, the two electrodes still show similar dominant EIS characteristics, including two depressed semicircles and the Warburg diffusion region in the low frequency region. Therefore, in order to directly compare the main impedance components of the two electrodes under a unified fitting framework, this study adopted the same equivalent circuit model. It should be noted that possible grain boundary and crack related resistive contributions in the pristine electrode are not explicitly resolved as separate elements in the present model but are mainly reflected in the fitted *R*_f_ and *R*_ct_ values. Physically, the high- and mid-frequency arcs are attributed to the CEI film resistance (*R*_f_) and the interfacial charge-transfer resistance (*R*_ct_), respectively. Meanwhile, the *Z*_W_ component reflects the intra-particle lithium-ion diffusion kinetics. Based on the equivalent circuit fitting, the total interfacial resistance (*R*_f_ + *R*_ct_) values for the pristine NCM811 and N-LSTO2 electrode after the 1st cycle are 112.77 Ω and 71.37 Ω, respectively. After 300 cycles, the values for both samples increase significantly. In particular, the total interfacial resistance values of the pristine NCM811 increases drastically to 306.92 Ω, while that of the N-LSTO2 electrode exhibits a much smaller increment, reaching only 175.48 Ω. The significant increase in interfacial impedance in the pristine electrode is consistent with the serious crack formation observed in Fig. 6. Previous studies have shown that microcracks in the nickel-rich cathode will promote the penetration of electrolyte into the particles and continuously expose the fresh surface to the side reaction environment, resulting in the thickening of the CEI film and the continuous increase of interfacial impedance.^31–33^ This contrast demonstrates that the LSTO protective layer effectively inhibits the continuous thickening of the high-impedance CEI layer, which usually comes from the side reaction at the electrode/electrolyte boundary. Subsequently, the Li^+^ diffusion coefficient was calculated using the following equation:
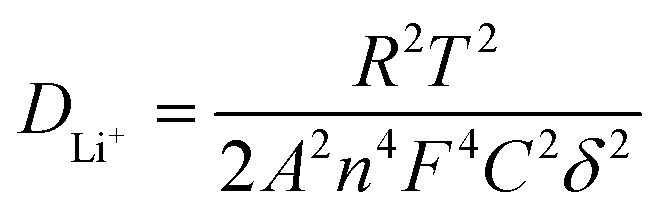
where *R* represents the ideal gas constant, *T* represents the absolute temperature, *A* represents the effective electrode area, *n* represents the number of transferred electrons, and *C* represents the concentration of the body phase Li. The Warburg factor *σ* is the slope of the straight line, which can be calculated by the relationship diagram between *Z*′ ∼ ω^−1/2^.^34,35^ Based on these parameters, the *D*_Li^+^_ values of pristine and N-LSTO2 samples are approximately 6.43 × 10^−12^ and 7.71 × 10^−11^ cm^2^ s^−1^, respectively. After 300 cycles, the *R*_f_ (82.78 Ω) of the N-LSTO2 electrode is much lower than that of the pristine sample (156.11 Ω). This result confirms that the LSTO protective layer effectively reduces interface polarisation by preventing the continuous accumulation of high-impedance CEI film during the long-term cycle. The improvement of this dynamics comes from two synergistic factors. First of all, as a strong physical barrier, the LSTO modified layer isolates the highly active positive pole from the liquid electrolyte. This spatial isolation inhibits harmful side reactions and promotes the formation of an extremely thin and dynamic CEI membrane. In addition, since Li^+^ (de)intercalation is a charge-compensating process dependent on simultaneous electron supply, LSTO layer with excellent electron conductivity builds an efficient surface electron transfer network. This rapid electron transfer eliminates the dynamic bottleneck of the interface, thus accelerating the overall Li^+^ diffusion dynamics in the bulk lattice. Based on the characterizations of XRD, HRTEM, EIS and CV, the LSTO coating layer forms a unique nanocrystalline-in-amorphous hybrid structure, which lays the physical foundation for its hybrid ion-electivity characteristics. The amorphous matrix breaks the long-range lattice restrictions and provides a seepage path to promote the rapid diffusion of Li^+^, as confirmed by the elevated diffusion coefficients. At the same time, the surface LSTO, as a local electronic transmission network, reduces electrode polarisation, which is validated by the minimized redox peak difference. By integrating these mechanisms, this hybrid LSTO coating layer can optimise the dynamic limitations of traditional inert coating layers to a certain extent.

To summarize the modification mechanism, Fig. 7c shows a schematic of the structural evolution during prolonged cycling. For the pristine NCM811 cathode (path A), the bare surface is directly exposed to HF etching from the liquid electrolyte. As cycling progresses, unmitigated lattice strain causes severe structural collapse and extensive microcracks.^36^ The electrolyte penetrates these cracks, triggering continuous CEI layer formation.^37^ In contrast, for the LSTO-coated NCM811 cathode (path B), the uniform LSTO coating acts as a robust physical barrier isolating the bulk material from HF erosion and side reactions. At the same time, its superior electronic conductivity builds an efficient electron-transfer network, facilitating rapid interfacial charge-transfer kinetics. As a result, a thin and stable CEI is maintained, and the mechanical integrity of the secondary particles is preserved even after 300 cycles.

## 4. Conclusion

In summary, we have constructed a robust surface structure for Ni-rich NCM811 cathodes, which featuring an amorphous LSTO coating layer. As a robust physical barrier, the modified surface effectively mitigates severe structural degradation and reduces the tendency toward phase-transition-related structural deterioration. Moreover, leveraging its superior electronic conductivity, the LSTO coating layer constructs a highly efficient charge-transfer network to facilitate interfacial kinetics, while simultaneously restricting the continuous growth of a highly resistive CEI layer during prolonged battery operation. Consequently, the optimal N-LSTO2 cathode exhibits a remarkable rate capability about 120.3 mAh g^−1^ at 10 C and an outstanding capacity retention of 88.19% following 300 cycles at 1C. This study clarifies the critical influence of multifunctional surface structural design on improving electrochemical performance.

## Author contributions

Song Sun: investigation, writing – original draft. Shengjie Zhou: investigation. Jiawei Wu: validation. Meixin Huang: formal analysis. Yu Li: supervision, writing – review and editing, funding acquisition. Sujiao Lan: writing – review and editing. Guanhua Yang: formal analysis, writing – review and editing, funding acquisition.

## Conflicts of interest

There are no conflicts to declare.

## Supplementary Material

RA-OLF-D6RA03778J-s001

## Data Availability

The datasets generated and/or analyzed during the current study are available from the corresponding author on reasonable request. Supplementary information (SI) is available. See DOI: https://doi.org/10.1039/d6ra03778j.
