# Supplementary material for: Enhancing the high-voltage electrochemical performance of LiNi0.8Co0.1Mn0.1O2 cathodes through La0.2Sr0.8TiO3 surface coating
Source: RSC Adv. 2026 Jul 28. Online ahead of print. doi: 10.1039/d6ra03778j (PMC13410997; doi:10.1039/d6ra03778j)
Supplement: RA-OLF-D6RA03778J-s001 [file RA-OLF-D6RA03778J-s001.pdf]

Supplementary Information (SI) for RSC Advances.
This journal is © The Royal Society of Chemistry 2026

Enhancing the High-Voltage Electrochemical Performance of $LiNi_{0.8}Co_{0.1}Mn_{0.1}O_2$ Cathode through $La_{0.2}Sr_{0.8}TiO_3$ Surface Coating

Song Sun[a], Shengjie Zhou[a], Jiawei Wu[a], Meixin Huang[a], Yu Li*, [a], Sujiao Lan[a], Guanhua Yang*, [b]

[a] Department of Food and Chemical Engineering, Liuzhou Institute of Technology, Liuzhou, 545616, P.R. China.

[b] Guangxi Key Laboratory of Automobile Components and Vehicle Technology, School of Mechanical and Automotive Engineering, Guangxi University of Science and Technology, Liuzhou, 545006, P.R. China.

* Corresponding authors: liyu-306@163.com (Yu Li); yghchem@163.com (Guanhua Yang)

Tab. S1 Rietveld refinement results of cell parameters of both samples

Sample	a (Å)	c (Å)	c/a	$I_{(003)}/I_{(104)}$	R_{wp} (%)	R_p (%)
NCM811	2.8739	14.2107	4.9447	1.2311	13.57%	9.21%
N-LSTO1	2.8742	14.2109	4.9443	1.2294	13.12%	8.56%
N-LSTO2	2.87805	14.2235	4.9421	1.2325	13.75%	9.72%
N-LSTO3	2.8766	14.2162	4.9420	1.2343	14.22%	9.87%

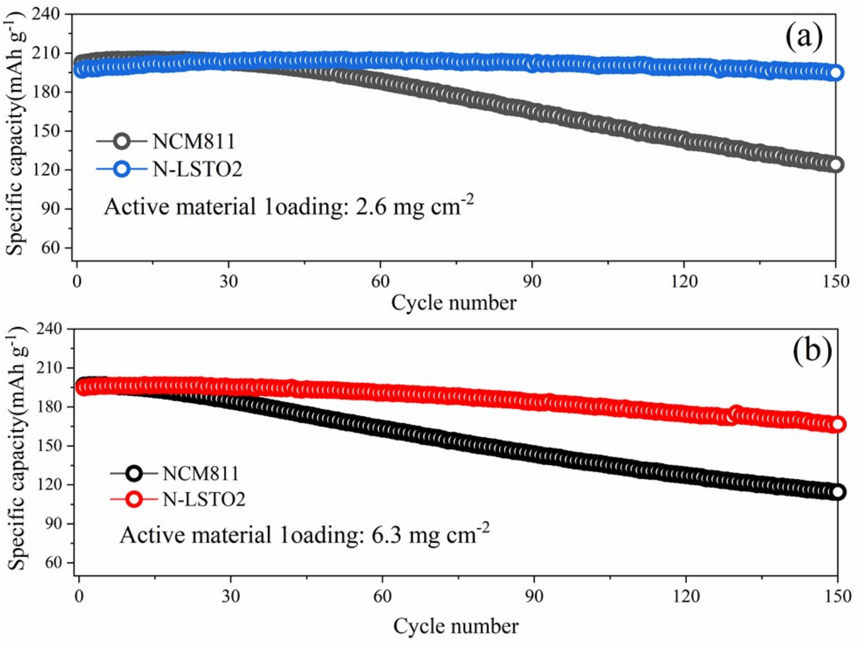

Fig. S1 Cycling performance of pristine NCM811 and LSTO-coated NCM811 cathodes under

different mass-loading conditions: (a) low mass loading (2.5-3.0 mg cm^{-2}) and (b) high mass loading (6.0-6.5 mg cm^{-2}).

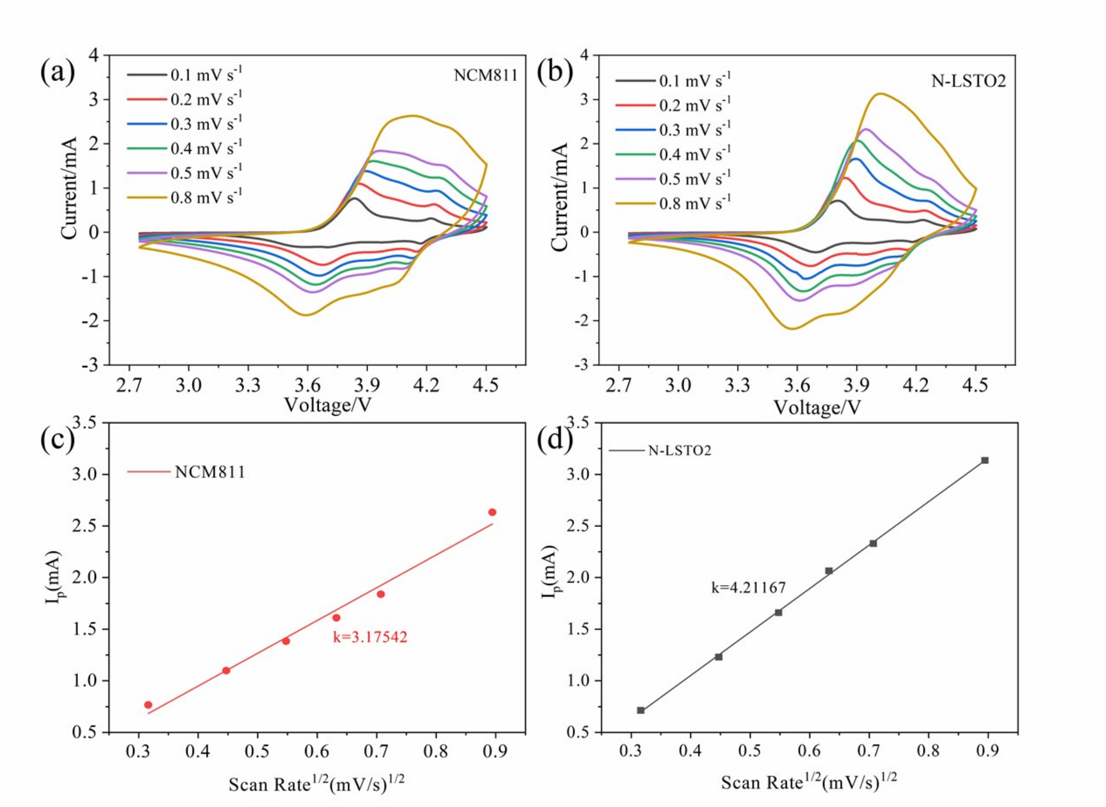

Fig. S2 Cyclic voltammetry curves at various scan rates from 0.1 to 1.0 mV s^{-1} for (a) the pristine NCM811 and (b) the LSTO-coated NCM811 electrodes. (c) The corresponding linear relationship and fitting slopes between the redox peak current (I_p) and the square root of the scan rate$^{1/2}$ ($mv^{1/2}$).

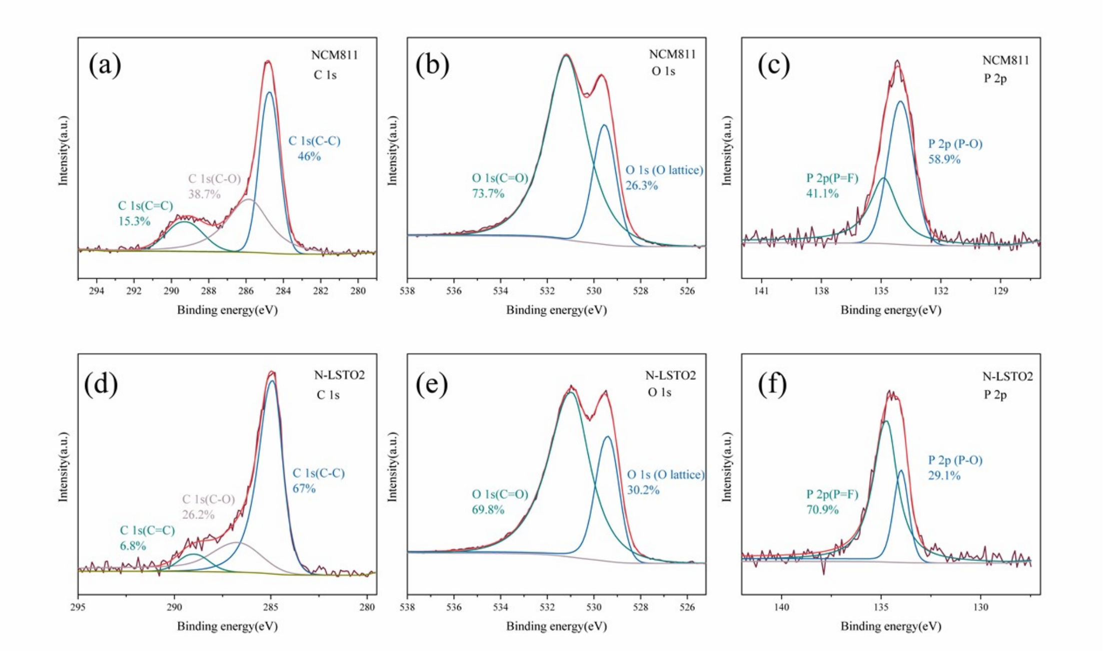

Fig. S3 High-resolution XPS spectra of the pristine NCM811 and N-LSTO2 electrodes after 150

cycles: (a, d) C 1s, (b, e) O 1s, and (c, f) P 2p spectra.

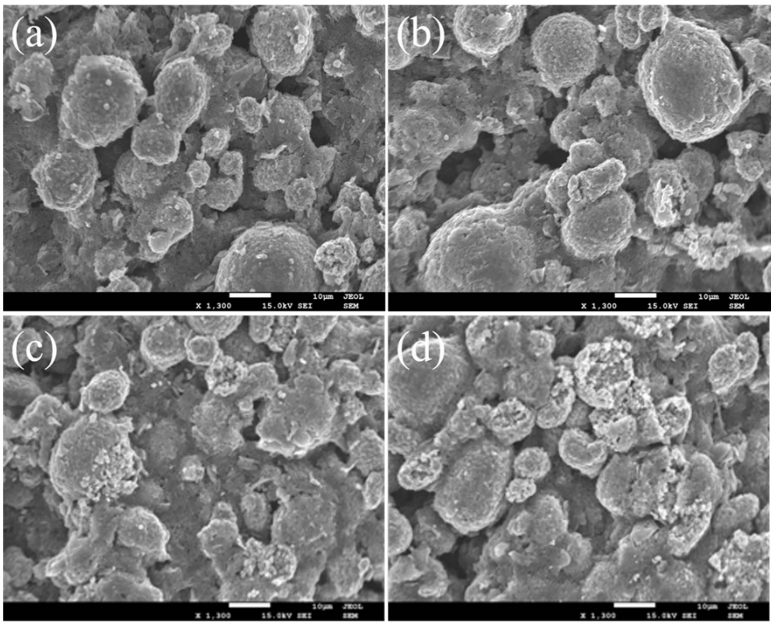

Fig. S4 SEM images of pristine NCM811 electrodes after (a) 0, (b) 50, (c) 100, and (d) 150 cycles.

Table S2 Comparison of the electrochemical performance of representative coated NCM811 cathodes reported in recent literature (2022-2026).

Coating material	Initial discharge capacity at 0.1C (mAh g^{-1})	Cycle condition (1C)	Capacity retention (%)	Reference
LSTO	204.7	300 cycles at 1C	88.19	This work
PLiMTFSI	156.42	100 cycles at 1C	86.0%	[1]
mesoporous SiO_2	210	100 cycles at 1C	87.3%	[2]
BTJ-L	173	100 cycles at 1C	86.1%	[3]
$LiAlO_2/Li_2SiO_3$	180.8	100 cycles at 1C	92.6%	[4]
$Co_3(PO_4)_2$	213	100 cycles at 1C	89.5%	[5]
TS-SN	210.2	50 cycles at 1C	90.4%	[6]
cPAN/ZIF-8	209.66	100 cycles at 1C	89%	[7]

[1] H. Kim, M. K. Jeong, H. J. Kim, Y. Kim, K. Kang, J. H. Oh, Small, 2025, 21, e2502816.

[2] M. A. M. M. Al-Samet, E. Burgaz, Electrochim. Acta, 2024, 507, 145167.

[3] Y. S. Wu, Q.-T. Pham, C.-C. Yang, C.-S. Chern, L. M. Babulal, M. Seenivasan, J. Jeyakumar, T. H. Mengesha, T. Placke, G. Brunklaus, M. Winter, B. J. Hwang, ACS Sustain. Chem. Eng, 2022, 10, 7394-7408.

[4] X. He, H. Luo, K. Yong, M. Yao, Y. Zhang, J. Energy Storage, 2023, 70, 1077872.

[5] T. Kim, M. S. Goh, H. Moon, H. Shin, J. Lee, H. Jeong, S. W. Joo, Y. S. Kim, Y. Im, M. Kang, J. Colloid Interface Sci, 2024, 670, 729-741.

[6] S. H. Kim, N. M. Shinde, Y.-E. Yun, J.-S. Kim, Batteries, 2024, 10, 453.

[7] J. Wang, J. Wei, D. Liang, L. Hao, Q. Zhang, H. Zhang, X. Li, Electrochim. Acta, 2026, 546, 147755.
